# Sharing Data – Not With Us! Distrust as Decisive Obstacle for Public Authorities to Benefit From Sharing Economy

**DOI:** 10.3389/fpsyg.2020.576070

**Published:** 2021-01-20

**Authors:** Ann-Marie Ingrid Nienaber, Andree Woodcock, Fotis K. Liotopoulos

**Affiliations:** ^1^Centre for Trust, Peace and Social Relations, Coventry University, Coventry, United Kingdom; Affiliated to the Centre for Management, University of Muenster, Muenster, Germany; ^2^Faculty Research Centre for Arts, Memory and Communities, Coventry University, Coventry, United Kingdom; ^3^SBOING, Thessaloniki, Greece

**Keywords:** distrust, sharing economy, public authority, crowdsourcing, community distrust, data sharing, transport and mobility sector

## Abstract

Future mobility planning to cope with ongoing environmental challenges such as air pollution has to be anchored in the work of every public authority worldwide. One recent trend that could support public authorities to meet the European Union’s sustainability targets is the creation and sharing of transport and mobility “big” data between public authorities via tools such as crowdsourcing. While the benefits of the use of big data to increase public authorities’ efficiency and effectivity and their citizens’ lives is well understood, examples from the public sector that highlight public authorities’ engagement in such sharing activities is still missing. To date relevant literature has highlighted issues around the capacity of public authorities that hinder shared activities. In this paper we want to raise distrust as a key reason for lack of engagement. Based on comprehensive data collected over the period of 4 years via several workshops and semi-structured interviews with seven public authorities in Europe, we are able to demonstrate that a major obstacle for not providing and sharing data via crowdsourcing for mutual benefit lies primarily in the hands of the public authority’s servants of the middle and high-level management. Our results show firstly, that distrust may emerge toward different referents such as the community, particular individuals, or the technology itself and thus, managerial implications have to be very specific to overcome distrust. Secondly, we show how distrust may spread from one referent to another through negative reciprocity and which, if unchecked may lead to an all-encompassing state that affects the whole sharing economy framework and inhibits potential benefits.

## Introduction

Public authorities are faced with a variety of challenging requirements from their citizens, international institutions (e.g., World Health Organization) and a diverse range of organizations caring about human health and well-being. Ambient air pollution for example contributes to 7.6% of all deaths following recent studies by the World Health Organization (WHO, 2016). The recent lock down due to the COVID-19 pandemic demonstrates an impressive reduction in air pollution’s due to less freight activities.

One of the key contributors to air pollution in Europe is urban freight activity which is increasing with the global rise of e-commerce and online purchasing. Delivery patterns and activities become more diverse, as products are increasingly delivered directly to the consumer, in addition to the classic goods’ distribution to local markets (Diana et al., 2018). Public authorities therefore need to find ways to deal with the European Union requirements by defining urban freight plans to study measures related to the efficiency of urban logistics and reduction of greenhouse gas emissions. One much hyped, recent trend that should support public authorities to meet the European Commission’ requirements is the use of “big data” (e.g., European Commission, 2020). Big data is described in terms of volume, velocity, and variety (Laney, 2001). It could foster a public authority’s efficacy, efficiency, and overall satisfaction by significantly increasing the accuracy of decision-making, accelerating organizational performance, and reducing operating costs related to the decision-making process. Using “big data” is not new, as there has been a long tradition in the production of very large datasets, such as national censuses, government records, and geometric surveys that provide information about cities and their citizens.

The public sector is aware that increased data availability and new methods of using it may be utilized for the public benefit (e.g., Manyika et al., 2011; Fazekas and Saussier, 2018). As such public authorities in many countries have adopted big data strategies or policies (UK Department for Business Innovation and Skills, 2013; US Executive Office of the President, 2014; for Europe see e.g., https://www.open-contracting.org/2015/06/02/digiwhist_big_data_meets_the_concerned_citizen/). However, unlocking the full potential of big data in the public sector requires public authorities to provide their data to open platforms and thus, to share their collected data with other public authorities and potential providers which have the ability to analyze and combine huge amounts of data sets to reveal trends and insights, which could be used by public authorities to probe new measures in transport and other domains. Following Cingolani et al. (2015) several European countries such as Italy, Norway, Poland, and the United Kingdom already publish fully machine readable public procurement data with other European countries moving toward this (Cingolani et al., 2015).

While it seems that the benefits of big data are well understood in relation to not only increasing a public authority’s efficiency and effectiveness but also to enhancing its citizens’ lives, examples from the public sector are missing that highlight public authorities engagement in such sharing activities. In this paper we focus on crowdsourcing as a platform for open collaboration, which is dependent neither on dimensions nor on geographical area. Collecting data by crowdsourcing has become a key stream to provide a smart way of resolving problems based on big data, collected by local authorities in relation to the community. The question to be asked, is why public authorities are still disinclined to engage in data sharing activities, when the technologies such as crowdsourcing platforms are in place already to share data with each other and to allow analyses, which will improve their organizational efficiency; lead to better plans and transport measures; provide evidence for investment; and measures which increase citizens’ health and well-being and increase the attractiveness of their cities.

While the literature highlights issues around legacy data sets and traditional ways of working (e.g., in siloes), and the capacity of public authorities that hinder public authorities from undertaking and benefitting from such activities (e.g., updated policies on sharing data by the European Commission in February 2020^1^), we want to shed light on the psychological effects of these uncertainties for public servants and provide a more nuanced and in-depth view on the obstacles for public authorities within our study group. We assume that one major driver for not providing and sharing data with other public authorities may lay in the hands of the individuals, i.e., middle and high level public servants’, driven by negative expectations toward different actors involved in the sharing economy, such as the service provider, other community members, or the technology itself beside external uncertainties.

Several scholars highlight that trust is the “currency” of a collaboration that includes the sharing of factors such as information (Botsman and Rogers, 2011; Mittendorf, 2018) in particular in relation to sustainability (Penz et al., 2018). The research notes that trust is a significant and decisive factor for successful collaboration and motivation to share knowledge or cooperate with stakeholders (e.g., Dyer and Singh, 1998; Nienaber et al., 2015; Brattström and Bachmann, 2018). Regarding sharing economy literature, researchers (Mittendorf, 2018; Penz et al., 2018) demonstrate that trust in platforms such as crowdsourcing is one of the main drivers for people engaging in online platforms.

Although trust plays a major role in sharing economies, there is little research on distrust and public management. Distrust is defined as the negative expectations toward another party’s intentions or behaviors (Van de Walle and Six, 2014). Recent findings by Strohmaier et al. (2019) or Gerber and Hui (2016) show that distrust plays a decisive role regarding business models such as crowdfunding or -sourcing. In particular, Strohmaier et al. (2019) highlight four different actors which can be the individuals’ target of distrust: interpersonal distrust toward the creator, organizational distrust toward the platform, institutional distrust related to the mechanisms and rules of the platform, and dispositional distrust toward the other users. Similar findings can be drawn from research on distrust and online platforms or technologies (e.g., McKnight and Choudhury, 2006) who argue that distrust is an additional factor that influences the users’ intentions to adopt a technology or not and is typically driven by different referents of distrust such as individuals, technology, service provider, and the wider system. Further, Nienaber et al. (2021) identified distrust as a key obstacle for the acceptance of new technologies in public authorities, also differentiating between individual, organizational, and system-level distrust. However, public authorities’ unease and distrust in data sharing has received little attention. Therefore, this study contributes to research by highlighting the decisive role of distrust in sharing economies built up of public authorities in Europe.

Using comprehensive data collected through semi-structured interviews and workshops with city representatives and wider stakeholders from seven European cities over 4 years as part of the H2020 CIVITAS SUITS project, this paper explores the role of distrust in contributing to the unwillingness of public authorities to share their city wide mobility data (and other forms of data, e.g., land use and public health) on platforms that would allow transport suppliers to unlock the full potential of big data to create significant benefits to public authorities. Supporting Urban Integrated Transport Systems (SUITS) is a 4-year research and innovation project, aiming to increase the capacity of small to medium cities to plan and implement sustainable mobility measures. During the project, seven cities [Kalamaria (Greece), Valencia (Spain), Alba Iulia (Romania), Rome and Turin (Italy), West Midlands (United Kingdom), and Palanga (Lithuania)] embarked on a journey that involved knowledge building, organizational change, and sharing of transport data on an online platform, provided by a private company. It was the service provider’s intention to run analyses on the data, analyse trends across data sets, etc., which could be of benefit to all parties by illustrating, for example, flows of transport using legacy data to predict mobility plans and improve transport related decision making.

The paper is structured as follows. First, a brief theoretical overview of the key aspects is given such as sharing economy and distrust. Secondly, the case study approach and the sample are described and results discussed. This paper finishes with managerial implications and a conclusion.

### Theoretical Background

#### Sharing Economy and Crowdsourcing

Today’s “sharing economy” stems from the confluence of several trends and technological changes (e.g., Cao, 2020; Sedkaoui and Khelfaoui, 2020). Growing ecological consciousness has encouraged people and organizations to share data for mutual benefits. The “sharing economy” is an umbrella term referring to the practices of sharing, exchange or rental of goods, services or knowledge to others through information technology, usually without the transfer of ownership (Taeihagh, 2017; Sutherland and Jarrahi, 2018). The sharing economy promises to increase efficiency and effectiveness by reducing transaction costs and supporting the creating of new knowledge by combining different views or sources of knowledge to support solution finding. As “An economic model based on sharing underutilized assets from spaces to skills to stuff for monetary or non-monetary benefits” (Botsman and Rogers, 2010), the sharing economy has established a new way of thinking and utilizing resources in an effective, efficient manner (Sutherland and Jarrahi, 2018).

The sharing economy is supported by crowdsourcing. This is defined as “the act of taking a job traditionally performed by a designated agent (usually an employee) and outsourcing it to an undefined, generally large group of people in the form of an open call.” Thus, crowdsourcing is the IT-mediated engagement of crowds for the purposes of problem-solving, task completion, idea generation, and production (Howe, 2006; Brabham, 2008). Crowdsourcing encompasses various types of platforms, such as virtual labor markets, tournament crowdsourcing, and open collaboration, which each have different roles and characteristics (Estellés-Arolas and González-Ladrón-de-Guevara, 2012; Prpić et al., 2015).

Crowdsourcing privileges a model of open collaboration, which is dependent neither on dimensions nor on geographical area. H. van Ess (2010) affirmed that “crowdsourcing is channeling the experts’ desire to solve a problem and then freely sharing the answer with everyone.” Collecting data by crowdsourcing has become a key stream to provide a smart way of resolving problems based on big data, collected by the community.

Big data, and methods for their use, are an emerging phenomenon in the management landscape that allows for an increasing efficiency and efficacy (Jabłoński and Jabłoński, 2020). However, unlocking the full potential of big data for the public sector requires a public authority to provide its data on open platforms and thus, to share their collected data with other parties and potential providers that help to analyze the huge amount of data sets to allow the creation of benefits to every party.

In this paper we focus on sharing economy on transport and mobility data built by different public authorities in Europe. All the public authorities shared particular characteristics, e.g., they belong to the public sector, face challenging agendas in their regions to cope with future mobility targets and need to demonstrate their engagement to support citizens’ health and well-beings in their regions. In addition, as members of the H2020CIVITAS SUITS project, each had committed to share mobility data to understand and find solutions, not only to their mobility problems, but in ways which would lead to greater understanding of the needs of small to medium sized local authorities across the EU. Each local authority was expected to share their knowledge in the form of data related to mobility on a test platform. The data would then be analyzed by a third party – service provider – to show how future mobility requirements could be managed in a more efficient and effective manner. The challenges of dealing with real-time traffic data collection and exploitation so far are manifold: firstly, even if data exists in a public authority, such data may be collected by different systems, from heterogeneous sources and in different formats, that makes them difficult to be integrated, homogenized, or correlated, secondly public authorities may not be aware of the relevance of that data, and the possible ways to use it and benefit from it, thirdly, it costs a lot to get it from third parties, fourthly the collection methodology is not sustainable, and finally, the data is not owned or controlled by the city.

#### Distrust and Its Evolving Process

Although traditionally there has been a paucity of research related to distrust (e.g., Rousseau et al., 1998), more recent publications show it is gaining interest (e.g., Bijlsma-Frankema et al., 2015; Guo et al., 2017; Haugsgjerd and Kumlin, 2020). Distrust is defined in several ways, either in contrast to trust (e.g., Lewicki and Tomlinson, 2003), or as a distinct concept to trust with different antecedents and outcomes (Lewicki et al., 1998, 2006). Recently, Bijlsma-Frankema et al. (2015, p. 3) defined distrust as: “a psychological state, comprising the unwillingness to accept vulnerability, based on pervasive negative perceptions and expectations of other’s intentions, or behaviors.” In this study, we align with this definition and agree with other scholars in the field that trust and distrust may co-exist and be simultaneously experienced as low or high (for an overview see Sitkin and Bijlsma-Frankema, 2018).

Scholars highlight different manifestations of distrust in the literature such as wariness (Lewicki et al., 1998; Chang and Fang, 2013), concern (McKnight et al., 2004), suspicion (Deutsch, 1958; Kramer, 1994; Lewicki et al., 1998), or message questioning (Sitkin and Stickel, 1996). Furthermore, parties that are not perceived as trustworthy are described as not competent (Sitkin and Bijlsma-Frankema, 2018) or as not fair and benevolent (Lind, 2018).

Distrust is mainly driven by attributions of negative motives (see Bijlsma-Frankema et al., 2015). Attribution is the process through which people try to explain their own and others’ behaviors (Heider, 1958; Kelley, 1967; Abramson et al., 1978). The proposed relation between distrust and motivational attributions is built on the notion that individuals feel the urge to interpret behaviors of others that are salient to oneself, such as harmful behaviors. The attribution of negative motives is a determinant of distrust when one party considers the motives of another to fundamentally and negatively affect a relationship through harmful behaviors or to provide the potential for harm (Sitkin and Roth, 1993; Bijlsma-Frankema et al., 2015). While the attribution toward motives might be related to a particular party such as a person or organization and thus, refer to historical events, value incongruence encompasses negative expectations toward the future (Sitkin and Roth, 1993). Perceived value incongruence is defined as “the belief that others adhere to values that are perceived as incompatible with the actor’s core values” (Bijlsma-Frankema et al., 2015, p. 1020). Based on the attributions, actors form deeper interpretations of the other party’s values as underlying mechanisms that produce their negative perceived behaviors. Indeed past research highlights the role of perceived value incongruence as a key factor in conflicts and conflict escalation in general (Sorensen and Sorensen, 1974; Pruitt and Kim, 2004; Fiol et al., 2009) and in distrustful relations in particular (Sitkin and Stickel, 1996; Chambers and Melnyk, 2006; Tomlinson and Lewicki, 2006; Bijlsma-Frankema et al., 2015).

The challenging aspect of distrust is its self-amplifying nature based on the negative attribution of motives and negative perceptions of behavior. Distrust will be expressed by manifestations such as concerns, skepticism, or worries. These manifestations and related behaviors demonstrate distrust in return, creating a pattern of reciprocal negative behaviors (Serva et al., 2005). Following the findings in conflict literature (e.g., Gouldner, 1960; Youngs, 1986), this process is reminiscent of the intensifying mechanisms in conflict escalation. If negative reciprocity develops, behaviors and behavioral reactions of the different parties involved become more harmful. The more a party’s negative behavior is perceived as harmful, the more negative the attributions. The following process of negative reciprocity fosters repetition of negative behaviors and expectations. Over time this spiral of emerging distrust merges into an all-encompassing stage of distrust including all parties involved. This process fosters perceived value incongruence of all involved parties and thus, hinders any collaboration between the parties in the future. Typical outcomes are the avoidance of interaction informally and formally a diminishing cooperation (Bijlsma-Frankema et al., 2015).

In our study, we focus on the public authorities as key members of the sharing economy and the private company that provides the crowdsourcing platform and runs the analyses. We seek to understand what reasons may have to be considered that hinder public authorities’ decision makers to share their data with the other public authorities and the service provider for mutual benefit. While bureaucratic issues such as required permissions from different institutions and missing clarification of the data value might have been obstacles for sharing data with others, we further assume that the public authorities are not willing to share their data on the platform to allow mutual benefit because of distrust toward different referents (i.e., actors).

## Materials and Methods

A case study approach was adopted that allowed us to enable the exploration of potential explanations for not sharing transport and mobility data between the public authorities via crowdsourcing (Yin, 2014).

### Sample

Data was gathered during intensive cooperation with the public authorities of seven European cities and their wider stakeholders in the H2020 CIVITAS SUITS project. Supporting Urban Integrated Transport Systems (SUITS) is a 4-year research and development project, aiming to increasing the capacity of small to medium cities to plan and implement sustainable mobility measures. The project addresses the ongoing major transformations in the transport sector which requires public authorities to work in new ways, with new partners, regulations, new modes of transport and notably, with innovative information and communication technologies. In the course of the project, the seven cities of Kalamaria (Greece), Valencia (Spain), Alba Iulia (Romania), Rome and Turin (Italy), Palanga (Lithuania), and West Midlands (United Kingdom) have embarked on a journey that involved knowledge building, organizational change, and sharing of transport data on an online platform, provided by a private company. It was the service provider’s intention to run analyses on the data, analyse trends across data sets, etc., which could be of benefit to all parties by illustrating, for example, flows of transport using legacy data to predict mobility plans and improve transport related decision making.

Thus, this collaboration of partners within SUITS has been forced by the project team. Although the different stakeholders signed a mutual agreement of working together over the period of 4 years to achieve the project’s targets, most of them did not know each other before as they have not worked together in the past. Due to this, the project team spent enormous effort in building trust between the different partners from the very early beginning. A three way engagement strategy was developed that was driven by (a) a series of interactive workshops to get to know each other and to exchange experiences and opinions, (b) an online forum for knowledge exchange, moderated by the project team, and (c) cross-learning sets between the local authorities to allow for individual and regular communication with each other.

The data for this study was collected through semi-structured interviews and 11 workshops with city representatives and through documentary analysis on individual’s perceptions and feelings when fostered to share data with each other.

In total we ran 18 semi-structured interviews with the key contacts in the seven European local authorities, we partnered with. All of these 18 participants belong to the transport or mobility departments in their local authorities and were heavily involved in SUITS. In detail 4 of them belong to Kalamaria (Greece); 2 to Valencia (Spain); 2 to Alba Iulia (Romania); 3 to Rome and 2 to Turin (Italy), 2 to Palanga (Lithuania) and 3 to West Midlands (United Kingdom). Of these 18 participants 13 are male and 5 female. All participants have a long work tradition in the public sector and on average worked for 9 years already in their local authority. All of the interviews were conducted in English. Participants’ basic English-speaking skills were determined during the initial discussion, once participants expressed an interest, and prior to the consent forms being signed. Additionally, to minimize language issues, all cities identified experts to support the interview partners. This was needed to ensure that all of our participants understood the questions correctly and were able to answer in the way they wanted without thinking about the “English” words when missing. Afterwards, we transcribed all the interviews in English and resent the transcripts to our interview partners for double-checking. Every interview took around 45–60 min.

The reason why we decided to conduct a semi-structured interview is that it has proved to be both versatile and flexible. It can be combined with both individual and group interview methods (Dicicco-Bloom and Crabtree, 2006) and the rigidity of its structure can be varied depending on the study purpose and research questions. The interview guideline comprised three main parts. In the beginning the participants were asked some demographic item such as gender, tenure to local authority, and work experience in the transport sector. Afterwards, we started to ask questions regarding their perception on the work collaboration between the partners in the project, putting emphasis on collaboration within the group, and the service provider in particular. Our questions were participant-oriented (Barriball and While, 1994) and not leading, and open-ended (Dearnley, 2005; Turner, 2010; Chenail, 2011) to generate answers from participants that were spontaneous, in-depth, unique, and vivid (Dearnley, 2005). This means that the answers reflect the interviewees’ personal feelings (Whiting, 2008) and perceptions which was mandatory for our study to understand perceptions of trust and distrust. The third part of the guideline was reflecting on factors that allow building of trust and reduction of distrust between partners in inter-organizational collaborations based on their experiences they talked about in the interview.

The 11 workshops were organized as a series of five different workshops plus two kick-off workshops driven by an engagement strategy for interaction and communication between the project team and the local authorities to foster trust building between the partners and focused on knowledge exchange between the seven European local authorities for mutual benefit. The six sequential workshops were held alternately in one local city or at a joint place to allow for specific learnings in one particular local authority in paying attention to the individual requirements driven by their organizational culture and history, and to foster knowledge exchange between the local authorities (i.e., experiences, best practice examples). The first two “Kick-off Workshops” included local authorities’ representatives from all cities to get to know each other and to build trust and mutual understanding. The second workshop included mainly local change agents, identified as local champions of change with a remit of: building and retaining trust as a means of reducing resistance to new technologies and ensuring a successful implementation. The workshop focused on how to promote safe learning cultures to enhance ideation, and the role of emotions in managing change and supporting how sense is made of experiences. The main goal of the third workshop was to establish local action learning sets and cross-local authority communities of practice learning sets. Workshop 3 brought together members and users from the different learning sets (developed in workshop 2) to meet face to face to facilitate the exchange of ideas and of support. These participants had an important role in sustaining change by offering fresh new insights from other contexts to enable challenges faced by one local authority to be overcome more effectively. Workshop 4 involved those from the second workshop reviewing their plans and the progress. It was a workshop designed to include reflection and review of progress, of mistakes and learning, and to focus on where to refresh and to reset different activities that are not working as intended. The last workshop focused on modeling a cycle of learning with emphasis on learning from others insights and adopting what worked, through insight not only into what to do but into why this bit is important in the transfer. While the jointly held workshops were attended by around 20–35 participants, in those workshops that were organized more locally with just three partners around 10 public servants attended. The key participants of the interviews were part of the workshops, too. All participants were always working in the transport or mobility departments of their local authorities. The majority of partners were male. The age varied between 27 and 60 years. The workshops were conducted by two members of the research team, who produced a report for each workshop. The reports were analyzed by a third member of the research team to establish a link between the workshop results and the results of the interviews. The objective of this approach was to increase the reliability of our results by using methodological and researcher triangulation (e.g., Bryman, 2013).

Beside these two sources of data, we also analyzed additional material we received from our partner local authorities related to their structural processes (hierarchy; communication flow) and general structures within the organization. This, documentary analysis examined documents such as minutes of meetings from the project’s steering group, departmental meetings, reporting sheets or work papers. This was important to understand the legal framework that played a major role regarding knowledge and in particular data sharing.

### Data Analysis

To analyze the data a thematic analysis has been used. Theoretical thematic analysis or deductive thematic analysis are mainly driven by a specific – theoretically underpinned – research question chosen by the researcher (Braun and Clarke, 2006). In this case, the data was coded according to *a priori* codebook, developed from a rigorous literature review on distrust. The objective of the analysis was to answer the key questions: “do public authorities’ employees (public servants) share their data via crowdsourcing?” and if not, “what are the reasons that make those employees struggle to share their data via such technologies?”

The data analysis followed three main steps in line with the requirements by Lee (1991) and Orton (1997) whose interpretive approach focuses on the integration of theory with organizational research. In the three-level model proposed by Lee (1991), opinions of the participants are validated and systematically transformed into theoretical understandings. We refrained from sorting the data into specific groups; instead we allowed data to be matched with theoretical concepts, so that new findings could emerge from the raw data (Lee, 1991; Bijlsma-Frankema et al., 2015). In the first level of analysis, we followed an inductive approach by comparing the data from the participants and discussing it in the context of the local authority to which they belonged. We were able to describe each local authority, its perspective on the inter-organizational collaboration for sharing data via crowdsourcing, and its relationships to the service provider separately. The second level involved sorting the data into broad second-order categories. For this, we analyzed the data step by step in relation to the different relationships in this collaboration between the cities, the SUITS team and the service provider. We sorted the data according to which aspects of the relationship were perceived as problematic or positive by both groups, the local authorities and the service provider. We further checked for the reasons behind the perceptions and looked for particular behaviors of the two groups that mirrored the perceived problem. In the third and last level of the analysis, the data was compared with ideas drawn from theory to build a theoretical understanding of the relations between the local authorities and the service provider. In accordance with Lee (1991), as we picked up hints indicating that distrust was a key component in most of the observed relationships between the local authorities and the service provider, the first set of analytical rounds determined whether the phenomenon observed between the partners was indeed distrust. Here, we checked the existence of actual distrust when requested to share data via crowdsourcing. The coding strategy was based on the literature on distrust to support the analysis of the interviews. The data was checked for key words related to distrust such as unwillingness to share, negative expectations, skepticism, concerns, fears, and negative feelings but also for the positive aspects such as trust, positive expectations, willingness to share. The second step of the thematic analysis comprised the identification of the reasons for employees’ distrust when sharing data via crowdsourcing. To do so, codes were considered for distrust and trust as belief and as behavior (Bijlsma-Frankema et al., 2015; Guo et al., 2017), as well as “trustworthiness” and “distrustworthiness” (e.g., incompetent, self-interested, exploitative, volatile, opportunistic). Data was coded at the explicit, rather than implicit, level, and results organized thematically, based on the patterns which emerged from the discourse (Deacon et al., 2007). In this way, we progressed from deductive “first-order codes” to inductive “second-order themes,” guided as appropriate by coding (such as that listed above) and thematic terminology found in similar studies (e.g., Bijlsma-Frankema et al., 2015). The findings section describes the most frequently found themes in relation to our research aim and theoretical framework. The third round of analysis tried to identify how distrust developed among the different partners.

## Results and Discussion

The first section of the results shows how we investigated whether employees’ distrust is actually present in the public authorities (step 1 of the analysis). The second section will highlight the process of how distrust is triggered and increased by negative reciprocity and promotes the pervasiveness of negative attributions that lead to an all-encompassing state of distrust that hinders any kind of sharing data via crowdsourcing (steps 2 and 3).

### Distrust Toward Four Different Referents in a Sharing Economy

In the interviews and meetings with the representatives from the public authorities, in particular the mobility and transport departments, it became clear that distrust was an issue for them not only in terms of cooperating with the crowdsourcing provider but also in sharing data with the other public authorities on the virtual platform. A variety of hints toward distrust were identified in our sharing economy. Key indicators were the hesitation and skepticism of the public servants toward the sharing economy framework. The diversity of the “distrust hints” led to the identification of different referents:

(a) the technology itself (crowdsourcing platform) called *system-level distrust*,
(b) the company as provider of the tool to analyze data (*organizational distrust*),
(c) the representative person/s of the service provider who was/were in direct contact with the public servants (*interpersonal distrust*) and finally,
(d) distrust toward the other public authorities sharing their data on the same platform (*community distrust*).

In the following we outline the different indicators for public servants’ distrust toward these four referents in detail, including examples from our comprehensive data.

(a) System-level distrust: In the first instance, it was striking that the public authorities did not show any interest in the benefits of sharing data with other public authorities. They did not try to understand the crowdsourcing tool or the analysis methods, ask any questions or respond to requests for some data, instead they simply expressed doubts as to the benefits of the approach. One quote for example to show this skepticism is “I am not familiar with that tool and I do not see the benefit of it except that I have to invest time and effort” [Public authority (PA) 4]. Another public servant said “what is new about the tool? I do not see any advantage for my city” [PA 3]. This skepticism or little interest that we were able to observe may have also been triggered by the fact that the public servants included in our study – although they have been public servants from the middle and high level – they were mainly from the mobility department and thus, they may be not directly benefiting from the sharing data system. Instead, they were facing a lot of work in collecting and sharing the data. Thus, the skepticism was very likely driven by a mixture of these reasons.

(b) Organizational distrust: “You said this tool does not cost us any money but I cannot see your business model then. What is your intention? What are you going to do with the data in the future?” [PA 1]. This statement highlights a variety of similar questions by the public servants that did not progress for 2 years. Even in the confines of a research project, the public authorities would not buy into this concept, just to see how it would turn out. They were distrustful of the suppliers’ business model as he was offering services without payment, in the hopes of generating use cases and testimonials. The public authorities remained skeptical about the supplier’s real motives and offered excuses such as “We do not have the data, another department is responsible for this data” [PA 2], or “we do not know really what kind of data is required” [PA5]. Based on these statements, we assume that the public authorities were not fully aware of the structures and processes behind the surface of this crowdsourcing tool, and thus, distrust the organization (underlying business model) as a whole instead of particular processes or structures.

At many stages there was a complete communication breakdown with the service provider just asking for any open data regardless of size, format, or currency, to test the concept and to actually make it easier for the public authorities to find the data. Potential obstacles related to confidentiality, General Data Protection Regulations (GDPR) or technological skills. Each party blamed the other for the impasse.

(c) Interpersonal distrust: This was exhibited in the nonverbal behavior of public authorities in the technical presentations, public authorities demonstrated their unwillingness to cooperate with certain people by, for example folding their arms, laying back in their chair, checking emails and messaging. The wariness and concerns regarding motivations was expressed during meetings at which these people were not present in comments such as. “He could not convince me at all. That was a really bad presentation that he gave and I cannot see any benefit” [PA 2]. “He was not able to present the tool at all, so how should I understand what they want to do with the data later? And when I asked him, he did not really answer my question” [PA 6].

(d) Community distrust: Beside these three referents of distrust another dimension of distrust was present, i.e., that between the group of public authorities themselves. While they all understood the relevance of sharing data with each other to be able to profit from each other’s experiences, levels of distrust were identified. “We expect that we have little in common with the others [public authorities] and that the others [public authorities] intentions are different to ours in the long run. Maybe that can harm us sometime” [PA4]. Thus, due to the sharing economy, a fourth referent for distrust has to be taken into account when trying to understand why public authorities are not willing to share their data with each other. Although community distrust may be similar to Strohmaier et al. (2019) definition of disposition of distrust in the first instance, they can be clearly distinguished. Disposition to distrust is defined as “a person’s consistent propensity or unwillingness to rely on or be vulnerable to others (McKnight and Chervany, 2001) or as a person’s general suspicion about humanity (Sitkin and Roth, 1993; McKnight et al., 2004). Community distrust is not just a general suspicion about humanity but distrust toward a clearly defined group of partners.

In the following Table 1 a few hints for distrust toward the different referents are summarized:

**TABLE 1:** Quotes of “distrust” toward different actors.

Public authority*	**Referent**	**Statement**	**Distrust type**
6	Company	“I am concerned what they (company) will do with the data in the long run and thus, I do not want to provide data for this tool.”	Organizational distrust
3		“I do not believe in what they tell us, I am questioning their business model and […]. No company can exist when not earning money.”	
5	Person	“I cannot follow him, what is his motive? Does he want purely our data without ownership – I cannot belief him, how will he earn money?”	Interpersonal distrust
1		“He does not look very competent in my eyes, I am really concerned whether they are able to work with our data in the way that we will benefit in the end.”	
2	Technology	“What will happen with the data? What is the tool doing? I suspect that this technology is working properly. I cannot see it visualized – so how should I understand its’ benefit for us?”	System-level distrust
4	Community	“We expect that we have little in common with the other [public authorities] and that the others [public authorities] intentions are different to ours in the long run. Maybe that can harm us sometime.”	Community distrust

*The number indicates the public authority.

The finding that trust is related toward different referents has already been shown in relation to trust research (e.g., Fulmer and Gelfand, 2012) and thus, it seems to be less surprising to see that different referents of distrust exist. In particular in the field of online platforms, decisions to trust are usually connected to different referents, e.g., the provider (organizational trust), the person that is representing the providing company and gets in direct contact with the other party (interpersonal trust) and finally the online technology, i.e., crowdsourcing, which is called level-based trust (van der Werff et al., 2018). Also in recent studies on distrust, different referents are discussed. Strohmaier et al. (2019) for example highlight in their study on distrust and crowdfunding four different referents, which can bet the target of distrust: interpersonal distrust toward the creator of a crowdfunding platform, organizational distrust toward the crowdfunding platform itself, and dispositional distrust toward the other users. Similar findings can be drawn from research on distrust and online platforms or technologies (e.g., McKnight and Choudhury, 2006), Nienaber et al. (2021) which differentiates referents as individual, technology, service provider, and the wider system. Thus, it can be seen that the first three distrust referents have been already discussed while the community distrust is mainly related to the sharing economy framework. While the first three referents of distrust seem to be typical when it comes to technology that is involved between two parties in a business context, the latter referent of distrust has not often been mentioned in the literature so far. This kind of community distrust is mainly driven by the sharing community and the fact that the different public authorities involved in the crowdsourcing platform may perceive each other as competitors, e.g., “I am not keen to show all our data, we all want to achieve the same goals and bigger cities having more capacity to take the benefits” [PA 6]. The perceived competition between the public authorities is likely triggered by their needs to be involved in government grants, citizen’s ranking related to livable cities or the decision making of companies’ top management where to found a company.

### Distrust as Underlying Reason for Not Participating in a Sharing Economy

In the next steps (steps 2 and 3) we followed the procedures of a thematic analysis in explaining how distrust may emerge in this case study. Our results highlight how firstly, the uncertain times of the macro-environment fostered public authorities skepticism toward the service providing company’s business model. Based on the public servants’ hesitation, perceived by the company as negative behavior, we can show that negative attributions toward motives allow distrust to flourish. A repeated negatively perceived behavior strengthened the emergence of distrust between the parties. Finally, our results indicate that distrust toward one referent may be able to trigger to another referent and herewith, supports the emerging of distrust between all parties involved in the sharing economy. This all-encompassing stage of distrust in the end will diminish any cooperation between the parties due to perceived value incongruence.

### Macro-Environment: Uncertain Times for the Trustor

The reluctance on the part of public authorities to share data is strongly related their concerns over data security and privacy in general, and GDPR in particular. This was introduced at the start of data sharing discussions and no one was entirely sure of the system, and certainly did not want to jeopardize their organizations by providing data in contravention to GDPR. The procedures needed for the project took 12 months to complete. Although the kind of data, the data source and the responsible department for sharing this data was identified in the local authority from the very early stage of this project, we perceived high levels of uncertainty by our city partners. These uncertainties could also not have been solved when including the top management of the local authority into the discussions as the legal issues around data sharing are discussed almost Europe wide (see e.g., https://www.open-contracting.org/2015/06/02/digiwhist_big_data_meets_the_concerned_citizen/). Thus, all local authorities were generally very sensitive regarding this topic.

Regarding mobility data, legacy data has not been collected in a standardized manner in any authority as it can be collected for a specific purpose, e.g., new road layout or environmental sensors over certain times. Its usefulness to others was therefore questionable. It had also been collected for a specific purpose, as such partners were unsure as to where it could be repurposed. The lack of understanding and oversight of current data may have left public authorities feeling very weak and vulnerable which fostered their concerns. Indeed, public authorities do not seem to have a coherent data management plan, with data simply gathered and held by individual departments collected to inform their work.

Additionally a lot of mobility data, while not containing personal or sensitive data, has sufficient information embedded within it, which when combined with other information, may allow some degree of identification of the information providers. This was discovered during our pilot case study in Kalamaria in which real-time mobility data was collected. This showed the need for a lot of post processing to ensure anonymization of data collected automatically for vehicles. Lacking the skills and knowledge to do this could be an added perceived vulnerability for public authorities especially with the introduction of fines for organizations found to be in breach of GDPR. Thus, they erred on the side of caution.

### Negative Attribution of Motives That Foster Repeated Negative Perceived Behaviors

Our data indicated several aspects that may be summarized as attributions of negative motives related toward one of the four referents.

Most of the public authorities avoided interaction with the service provider since they did not believe the company’s business model. It is important to mention here, that this was not simply driven by the fact that due to the project SUITS the local authorities and the service provider did not share a long history of interaction with each other. Due to the engagement strategy, including workshops, formal and informal meetings, etc, and the role of the project management of SUITS, functioning as an intermediary to develop trust between the local authorities and the service provider, trust was built between the project partners. Thus, we are convinced that further factors triggered the emergence of distrust. First, offering their services voluntarily to the public authorities made them seen less trustworthy as the public authorities suspected their business model. “I do not really understand how this should help us and how we can benefit from it. Thus, what are the real motives behind this tool?” [PA 5]. Another public servant asked: “What is your business model? How do you get money out of that? I have to understand this, otherwise I cannot believe in you” [PA 1]. This result is rather surprising as the service provider was payed by the EU grant for developing this model, following the agreement, the public authorities were asked to share their data to allow the business model to be tested. However, the observations lead to the feeling that the service provider was really much interested in getting the data from the public authorities which made them rather distrusting the service providers’ motives instead of supporting the idea of sharing data on the crowdsourcing platform. The public authorities became worried about data breaches, exploitation of data, and data as a competitive and lucrative asset. A different emphasis on the original idea to generate a mobility product could have been more successful.

These statements indicated that the public authorities’ concerns were driven by uncertain macro-environment regarding the confidentiality of their data and the long-term use of the data once it is passed to a private company. The public authorities have a responsibility for the data they collect, and for the citizens who provide that data. Even in cases where anonymized data can be used to help develop better transport systems and services, public authorities are still wary. Negative press releases and data breaches were a major source for their concerns as these would have very negative consequences for the public authority as an organization but also may have negative consequences for those individuals that made the decision to share data on the platform. Distrust arises as others come to be characterized as unpredictable and threatening, thus fostering a sense of uncertainty and vulnerability (Sitkin and Roth, 1993). Although research shows that low levels of distrust are healthy and prevent individuals to make failures or trust “blindly,” the indicated level of wariness was relatively high (e.g., Lewicki et al., 1998). Thus, we assume that while trustful relationships between the local authorities and the service provider were established in line with the comprehensive engagement-strategy within the project, the perceived risks derived from the uncertain macro-environment overwrote the levels of trust toward the service provider (e.g., interpersonal or organizational level).

Furthermore, the poor marketing of the new technology by the service provider did not help. The representatives were not able to demonstrate a visualization of the tool, or a working system, they did not highlight its benefits for the public authorities, and did not try to talk to them in a way they would understand. As such it remained unclear how crowdsourcing would work in combination with the analysis tool, what the benefits would be (and for whom), who would use it and how it would integrate with existing systems. Transparent and clear answers were not provided. There might have been language and disciplinary barriers, and the developers lacked the interpersonal skills and knowledge needed to convince the public servants. Thus, there were still uncertainty about the functioning of the technology itself which triggered distrust on the system-level.

The skepticisms and concerns of the public servants showed on the one hand that they distrusted the competencies of the service company’s representatives (interpersonal distrust): “Sorry, I cannot believe your purely altruistic behavior” [PA 2] and on the other hand that they distrust the business model of the company (organizational distrust): “I do not really understand how this should help us and how we can benefit from it. Thus, what are the real motives behind this tool?” [PA 5].

Additionally, the technology itself was not trusted, with issues raised about data security and ease of use (system-level distrust). In the interviews and workshops the majority of public servants for example kept saying that they do not understand what kind of data format was needed although this point had been discussed several times. Furthermore, the public servants started to find excuses for their “not-cooperative” behavior, e.g., “our data is not retrievable” [PA 3] or “we have just a very tiny amount of data – probably not useful for this tool” [PA 1]. These comments are indicative of distrust (e.g., Sitkin and Bijlsma-Frankema, 2018). Other excuses for a “non cooperation” were for example: “I [PA 4] rather question whether this technology really works for us, you know, our local authority is very traditional and citizens expect us to take responsibility for taking care of them.” The latter examples demonstrates little connection with the original request.

While we could observe increasing trust between the local authorities and partners of the SUITS projects over the first time of the project, we notice some contradicting tendencies over the course of several meetings particularly related to the crowdsourcing tool and the request to share data, distrust emerged that became more and more pervasive, i.e., the public servants did not differentiate in their distrust of the service provider, its’ representatives or the technology. They were unwilling to expose themselves to vulnerability by sharing data with anybody, including their own community. One example that highlights this kind of community distrust is the following: “We expect that we have little in common with the others and the others intentions may different to ours in the long run. Maybe that can harm us sometime” [PA 4].” Part of this may be due to the increasing worries about data breaches, exploitation of data, and data as a competitive and lucrative asset.

Overtime we saw a build up of distrust, which the technology providers were not able to overcome, and thus spread across all referents, throwing into doubt the sharing economy as a viable concept. “They [company] do not really listen to what we tell them – we provided them a lot of information but actually they did not come back to us – so I assume we are not on the same page” [PA 3] highlights this rather all-encompassing state of distrust after a while. Thus, the idea of reciprocity as a driver of conflict escalation becomes obvious (Friedman and Currall, 2003; Pruitt and Kim, 2004). The self-amplifying circle of distrust, which we could identify, became the major obstacle for progress of this part of the project and any cooperation in the future where a sharing economy business model is not fully transparent.

The self-amplifying circle of distrust resulting in an all-encompassing state of distrust is built on the connection between negative attributions of motives toward each other based on negative experiences in the past and anticipated negative behavior in the future. The latter one turns into a general value incongruence between the involved parties (Bijlsma-Frankema et al., 2015). These perceptions of value incongruence between parties prohibit any future interactions or cooperation as the parties feel increasingly vulnerable when working with the other(s). Most authorities did not share their data, so the intended mutual benefit and exploration of the value added to the organizations of this approach was not investigated.

### Managerial Implications

Our results highlight three decisive implications for the practice of sharing economies. First, mutual benefit can only be achieved when distrust is not able to flourish. While little distrust might be helpful to clarify issues of legacy or responsibility and thus, increases mutual benefit in the end, high levels of distrust may prohibit sharing economy models. Potential causes of distrust have to be identified early enough to stop it triggering over into other referents. Thus, practitioners have to be mindful of any potential indicators of distrust to avoid further diffusion. As long as distrust affects only one referent, the literature on trust repair offers several options on how to overcome it using trust-building activities (e.g., Lewicki and Wiethoff, 2000; Tomlinson and Mryer, 2009). In this project we applied a comprehensive engagement-strategy to allow trust to be built at the beginning and over the lifetime of the project (i.e., several face-to-face meetings, regular email contact, and phone calls). The project management served as a mediator between the public authorities and the service provider trying to minimize any concerns. However, at that moment when distrust became prominent between the local authorities and the service provider, the project team initiated meetings either between the project management and the public authorities or the project management and the service provider to identify the most effective way to reduce distrust between the two parties, joint meetings were set up in a second step. Meetings were also organized by the project management to create a neutral platform for joint discussions. These face-to-face meetings and preorganization of joint discussions were most helpful to minimize the existing distrust levels.

One recommendation was to highlight more the benefit of the sharing economy model for the public authorities and to pay attention to the fact that those public servants that are providing the data might be not benefiting in the end. Thus, for them the data sharing process is mainly connected to higher workloads. Further, for highlighting technology’s functions a visualization is very useful to demonstrate its perceived ease of use and perceived benefit of use (e.g., McKnight and Choudhury, 2006; Nienaber et al., 2021).

Second, our results also indicated that an uncertain macro-environment triggers distrust. Our data highlighted that public servants need more clarification regarding the legacy and handling of data in their public authorities to be willing to make decisions and to take the responsible for these. During this time, negative expectations about the consequences of inadvertent mistakes in data handling were dominant and triggered distrust and risk aversion. High profile incidents regarding data misuse such as google analytics, made public authorities very skeptical and concerned when sharing data with relatively unknown organizations, i.e., private service providers. The public authorities’ lack of knowledge and insecurities over the value of their data, its ownership and possibility to identify citizens led to a lot of insecurities which the private company could not address. The failure by them, as experts to provide answers relating to big data, led to more negativity and distrust. Currently, public servants and other data managers do not share data and find it difficult to take the responsibility for making a decision regarding the release of a dataset as open data, which could put them in breach of GDPR, lead to data infringements and breaches, effect revenue streams, and cause reputational damage (e.g., through negative benchmarking). Furthermore, if a public servant makes the decision to share the data they may later on be accused of “not preserving the public interest” and “wasting public revenue.” The uncertainty of the macro-environment and potential consequences indicate that sharing data under these conditions is one of “blind trust.” Therefore, we state the external conditions under which a decision to trust or distrust has to be made, could trigger a “healthy” level of distrust which has to be overcome in the future to allow public authorities to benefit from sharing data with each other and further analysis to increase their efficiency and effectiveness to enhance citizens’ well-being.

Finally, we want to encourage all organizations and individuals that may want to initiate some form of crowdsourcing to share data to pay great attention to trust-building mechanisms and activities in the early beginning between the involved parties. Several scholars were able to demonstrate a variety of tools and activities to support a trustful climate, which allows a sharing economy to flourish in a way that all parties will benefit in the end (e.g., George, 2005; Maurer, 2010; Nienaber et al., 2015).

## Limitation

As in every study, some limitations have to be mentioned. First, in the course of this project, the seven local authorities have been forced to work together for 4 years according to the grant agreement which was signed by every partner. Thus, this collaboration of partners within SUITS has been forced by the project team. Although the different stakeholders signed a mutual agreement of working together over the period of 4 years to achieve the project’s targets, most of them did not know each other before as they have not worked together in the past. This created a rather “unnatural” collaboration between the partners as they were thrown together without a joint history of working experiences as it is typically the case in business collaborations. This fact of course limits the generalizability of our results to those collaborations which are driven by external circumstances such as “grant applications” or “requests by third parties.” However we want to raise two important issues that compensates this limitation. Firstly, this kind of collaboration is a typical phenomenon in the academic world and thus, our results can provide very useful insights and learnings for a great amount of future collaborations like this. Secondly, the project team spent enormous effort in building trust between the different partners from the very early beginning. A three way engagement strategy was developed that was driven by (a) a series of interactive workshops to get to know each other and to exchange experiences and opinions, (b) an online forum for knowledge exchange, moderated by the project team, and (c) cross-learning sets between the local authorities to allow for individual and regular communication with each other. These activities allowed trust building between the parties in a very rapid and strong way. Further, we have to confess that we only focused on crowdsourcing as a tool to share data with each other, provided by one service provider. Of course, future research is needed to compare the results of different case studies and to shed more light on further potential referents of distrust in such sharing communities and to provide useful insights about how to stop distrust to flourish in a community over time.

## Conclusion

With our study, we addressed an existing gap in research by analyzing the role of distrust when sharing data between local authorities and a service provider via crowdsourcing, applying a case study approach over 4 years. Based on comprehensive data, we were able to show firstly, that distrust in our study can be perceived toward four different referents and thus, needs to be specifically addressed during relationship building. Secondly, we were able to provide evidence on the idea of a self-amplifying circle of distrust driven by negative reciprocity. While distrust in its early stages is usually perceived toward one particular referent, distrust tends to trigger to other referents when it lasts for some time and emerges at higher levels. When distrust reached its threshold, our results demonstrate that distrust is able to take an all-encompassing state. This prohibits any kind of sharing economy such as our case study focusing on sharing data via crowdsourcing in the public sector. Herewith, our results complement the understanding of barriers regarding data sharing via crowdsourcing in the public sector with focus on distrust.

## Data Availability Statement

The raw data supporting the conclusions of this article will be made available by the authors, without undue reservation.

## Ethics Statement

The studies involving human participants were reviewed and approved by Coventry University’s Ethics Committee and European Commission Ethics Committee. The patients/participants provided their written informed consent to participate in this study.

## Author Contributions

A-MN served as the main author. She was leading on the development of the manuscript and responsible for collecting and analyzing the data this manuscript is based on. AW was leading on the whole SUITS project and supported Nienaber with her comprehensive knowledge in the field and her experiences when working with local authorities. FL was mainly responsible for the parts of the crowdsourcing aspect from the technological side. Liotopoulos is a practitioner and thus, contributed decisively to the understanding of the company’s side in this manuscript. All authors contributed to the article and approved the submitted version.

## Conflict of Interest

FL is the General Manager of the company (SME) named ‘SBOING’ (www.sboing.net). The remaining authors declare that the research was conducted in the absence of any commercial or financial relationships that could be construed as a potential conflict of interest.

## Footnotes

1 https://ec.europa.eu/digital-single-market/en/data-policies-and-legislation, updated February 2020.
